# My grandmother’s rug

**DOI:** 10.7554/eLife.109153

**Published:** 2025-09-22

**Authors:** Eve Marder

**Affiliations:** 1 Volen Center and Biology Department, Brandeis University Waltham United States

**Keywords:** living science, international collaboration, science, politics, None

## Abstract

The objects that migrants carry with them are tethers to the lives they have left behind.

I am aging together with the rug my grandmother made over 90 years ago. My father was born in Vienna in 1925, and eight years later, when my grandmother was pregnant with my uncle, she hooked a wonderful rug of bright blues, greens, yellows, and reds in a Turkish-inspired design. It is ~10 ft x 12 ft and now lives on my living room floor. I look at it every day, seeing in its fading colors and loss of definition a metaphor of my own aging.

The rug has its own immigrant life story, which is increasingly poignant in today’s world. In 1938, my grandfather announced that he and his family were leaving Vienna, before it was too late to survive the Nazi regime. They went with what they could carry, and locked the door, leaving behind the rug and the rest of their belongings. Nine months and many stories later, my grandfather managed to enter the US, followed by my grandmother, father and uncle. Like many other immigrants, they soon became naturalized US citizens. My brother, sister, and I were born in New York, where my grandparents settled.

Shortly after my immediate family left Vienna, other family members salvaged my grandparents’ most precious belongings and sent them to my grandfather’s shipping agent in Amsterdam. In 1946, when the war was over, my grandfather wrote to the shipping agent asking for the return of his stored goods. Several months later the rug and a set of silver candlesticks safely arrived in New York, having survived many years in Nazi-occupied Amsterdam.

My grandmother died a few years later, and the rug migrated to the living room floor in my parents’ then new house in 1958, where it was a major presence in our lives. When my father moved again, the rug took its place of honor in his living room. As my father grew old, the rug was rolled up and once again placed in storage. In 2008 when my husband and I moved into our new apartment on Boston’s waterfront, my then elderly father sent me the rug for my living room floor, where it has since been.

After several years I noticed that small regions of the rug were losing their vibrant color, and I realized that its proximity to the salt air was causing it to capture moisture, and the colors were bleeding, losing the definition of the design. The rug was becoming thinner, showing the wear from many years of use. I then faced the dilemma of whether I should continue to use and enjoy it, or to put it into storage to preserve it against the ravages of light and moisture. My father always said, when giving us new clothes: “wear it in good health and tear it in good health”. When I asked my siblings, they echoed similar sentiments, so our collective decision is that the rug’s value is in its use, not in its preservation.

Every day when I look at the rug I see additional evidence of its aging and loss of definition. Every day it reminds me of displaced families and political events that alter family history. As the rug ages in place, it reminds me of the extraordinary resilience shown by my grandparents, and by so many other enforced migrants then and now. It reminds me of the horrors of World War II, and of the survival stories from those days and today. When I was a postdoc, I met a Japanese-American man whose family lost their California farms and businesses when they were interned during World War II. And when I was in high school I watched economically disadvantaged classmates be drafted and sent to Vietnam, while many more privileged students avoided the draft.

**Figure:**
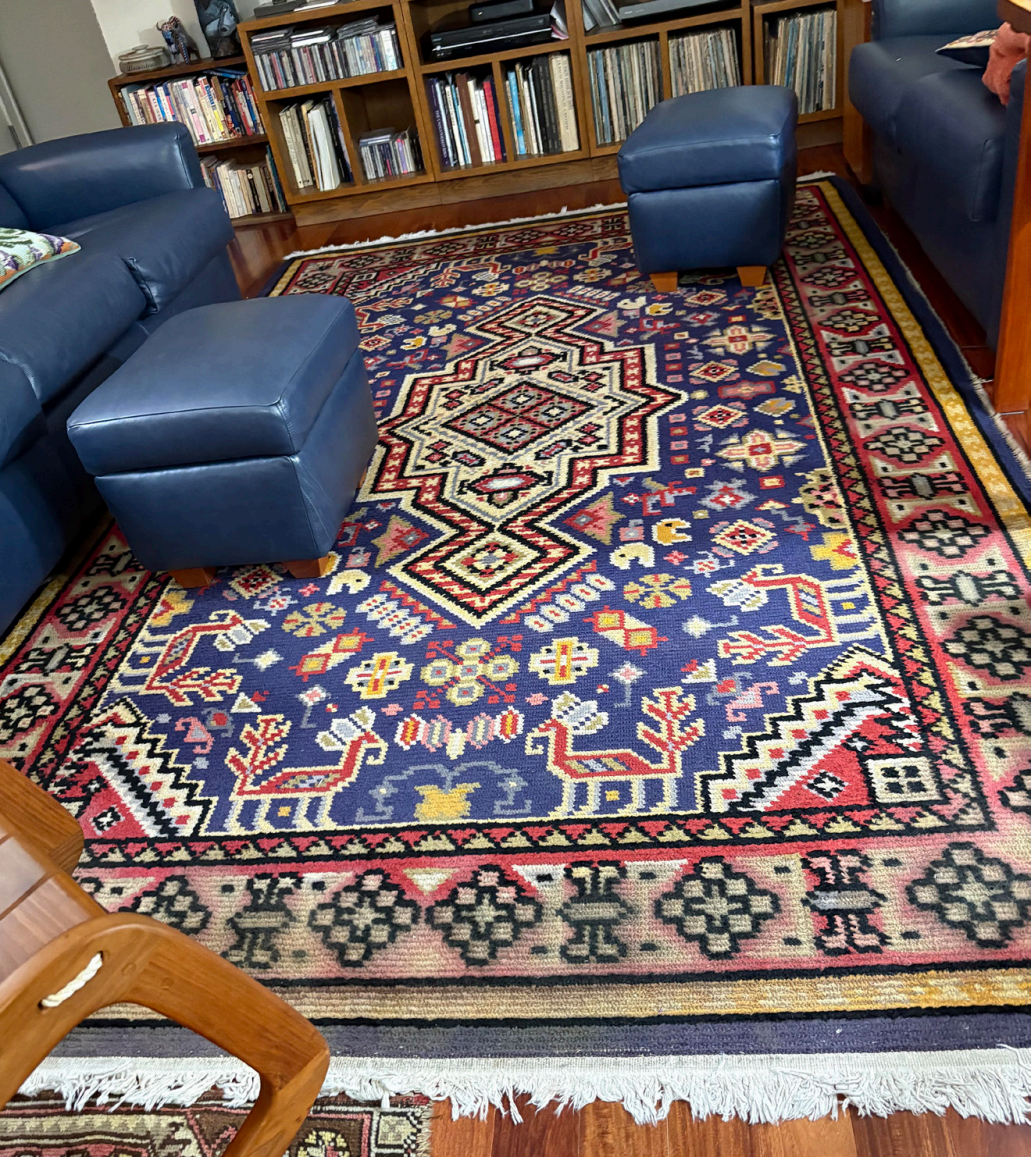
This rug was made by the author’s grandmother in Vienna in 1934. After the Nazis invaded Austria in 1938, the rug was moved to Amsterdam, which was invaded in 1940, and then to the United States in 1946.

Image credit: Eve Marder

Being sentient as an American has always meant acknowledging that US history is replete with shameful episodes, from slavery to the theft of land from our native populations, all accompanied by political and social hypocrisy. But this hypocrisy feels worse today than at any time in my personal memory, because it is not hidden and the scale and ruthlessness of the government’s actions against immigrant populations seem unparalleled in recent history.

Forced and voluntary migration has been an integral part of the international scientific enterprise, going back to the earliest days of modern science. My lab has welcomed people from across the world – from France, Germany, Iran, Chile, Argentina, Mexico, China, Korea, Belgium, the United Kingdom, Turkey, Ghana, Canada, Russia, Israel, Egypt, Romania, Jamaica, and Macedonia – including some who were emigrating in response to political duress. Lab alumni now live and work in many different countries, some of them returning home, and others, including Americans, having chosen to live elsewhere.

Much has been written about the damage being done to science, education and health care by the current US administration. Cut or delayed funding, disruption of the agencies that regulate therapeutic advances, and loss of respect for evidence are making it far more difficult to do science, and to educate the next generation of scientists. Science is difficult enough without visa and immigration nightmares, and policies that curb international mobility and collaboration now slow the discovery process at a time when scientific progress is critically important.

We all know that creating and building excellence, in science and other human endeavors, takes years of deliberate and intentional work, but excellence can be destroyed rapidly. It is unclear today how resilient American science and health care will be in the face of sustained attempts to discredit science and education. Presumably, as with many biological processes, the time-constant of recovery will depend on the duration and extent of the damage.

Experimental psychology and neuroscience have taught us that memory is reconstructive: the brain does not store a perfect snapshot of the past but, instead, uses logic and inference, along with the information it has stored, to recall past experiences. The loss of color and definition in my grandmother’s rug matches my own, less-than-perfect recollections. US science without international collaboration will be far more compromised than the gradual and graceful fading of my grandmother’s rug, and like degraded memory and loss of the rug’s color, may be irreversible.

## Note

This essay is part of the Living Science collection.

